# The Contemporary Role of Interventional Radiologists in Endovascular Aortic Repair: A Survey of CIRSE Members

**DOI:** 10.1007/s00270-025-04230-4

**Published:** 2025-10-15

**Authors:** Mohamad Hamady, Catharina S. P. van Rijswijk, Hicham Kobeiter, Maria Antonella Ruffino, Fabrizio Fanelli, Patrick Haage, Romaric Loffroy, Birgit Slijepčević, Stefan Müller-Hülsbeck, Gerard O’Sullivan, Florian Wolf, Robert A. Morgan

**Affiliations:** 1 Imperial College, St Mary’s Campus, London, UK; 2 Department of Radiology, C2-S, Leiden University Medical Center, Leiden, The Netherlands; 3 Radiology Department, H. Mondor Hospital, Assistance Publique-Hôpitaux de Paris, University Paris Est Creteil, Creteil, France; 4 Interventional Radiology, Institute of Imaging of Southern Switzerland - EOC Lugano, Lugano, Switzerland; 5 Vascular and Interventional Radiology Department, “Careggi” University Hospital–University of Florence, Florence, Italy; 6 Helios University Hospital, University Witten/Herdecke, Wuppertal, Germany; 7 Department of Diagnostic and Interventional Radiology, François-Mitterrand University Hospital, Dijon, France; 8 Cardiovascular and Interventional Radiological Society of Europe, Vienna, Austria; 9 Academic Hospital Christian-Albrechts-University Kiel, Kiel, Germany; 10 University Hospital Galway, Galway, Ireland; 11 Division of Cardiovascular and Interventional Radiology, Medical University of Vienna, Vienna, Austria; 12 St George’s University of London, London, UK

**Keywords:** Interventional radiology, IR, EVAR, Aorta, Endovascular aneurysm repair, Vascular

## Abstract

**Purpose:**

To describe the outcomes of a survey conducted among CIRSE members on endovascular aortic repair and to outline the future practice and needs of CIRSE members in this area.

**Materials and methods:**

An anonymous online survey with 21 questions was designed by the authors and sent to CIRSE members worldwide; data were collected for 12 weeks.

**Results:**

The survey collected 326 complete responses, with most respondents practicing in Europe. Two-thirds of respondents indicated that aortic repair is performed by multidisciplinary teams including IRs, with more than half reporting that they are equal partners in performing the procedure, and 27% acting as primary operators. In their practice, the most frequently performed endovascular aortic procedures were EVAR and embolisation of endoleaks, followed by FEVAR and TEVAR. A majority of 64% of respondents predict a growth in their endovascular aortic practice in the next 5 to 10 years that could further be supported by training, an increase in the workforce, societal guidelines, and additional qualifications. Hindrances for growth include turf competition as well as hospital management decisions.

**Conclusion:**

The survey indicates that Interventional Radiologists continue to play a significant role in endovascular aortic procedures and identifies challenges and opportunities to further increase the role of IR in endovascular aortic aneurysm procedures for the benefit of patients. The authors consider it essential for national societies as well as CIRSE to facilitate and encourage the clinical role of IRs in standard and complex EVAR to best guide future developments for optimal patient management in aortic disease.

## Introduction

Endovascular aortic repair is an established approach to treat infrarenal aortic aneurysms and is considered to be the first-line option for infrarenal aneurysms that meet anatomical criteria in several centres [1]. With the advent of branched and fenestrated stent graft techniques, even juxtarenal abdominal aortic aneurysms, thoracoabdominal, and aortic arch aneurysms can now be treated effectively via endovascular methods. Interventional radiologists have played a pivotal role in the initial development of this technique and continue to play a substantial role.

Several IR pioneers have contributed significantly to the development of endovascular aortic repair, serving as principal investigators or co-investigators in several landmark trials [2–9]. Although this expansion should make IR proud of their achievements, a gradual shift in practice has become apparent in the United States and Europe due to a variety of reasons.

This survey aims to evaluate the contemporary participation of IRs in endovascular aortic repair, to identify challenges, and explore strategies to boost the engagement of CIRSE members in this field.

## Purpose

To understand the current role of interventional radiologists (IRs) in endovascular aortic repair and to outline the future practice and needs of CIRSE members in this area.

## Materials and Methods

A comprehensive online survey was distributed by email among 7,290 CIRSE members and remained open online for 12 weeks to assess various aspects of interventional radiology (IR) involvement in endovascular aortic repair practice. The survey included 21 questions covering demographics, previous and current experience with aortic procedures, levels of participation in aortic repair (including primary procedures and follow-up interventions), and the training provided to junior staff. Display logic software was included that automatically triggered follow-up questions based on answers provided. Additionally, the survey explored reasons for any lack of engagement and potential strategies to strengthen IR’s role in this field, as well as the role of CIRSE in providing additional support if needed. Participants were allowed to submit open-ended comments regarding the types of assistance CIRSE might consider supporting members to improve the clinical management of aortic patients (see Table 1). The responses were collected anonymously in an online survey tool (Alchemer LLC, USA) and analysed using Microsoft Excel 365 (2024, Microsoft Corporation, USA).

**Table 1:** Questions–CIRSE member survey on endovascular aortic repair

General Demographics *1) Where is your centre located? [country dropdown]* *2) Please indicate your age group:* ( ) Below 35 years ( ) 35–45 years ( ) 46–55 years ( ) Above 55 years *3) What type of centre do you work in?* ( ) General/public hospital ( ) Teaching/University hospital ( ) Private hospital/clinic, foundation, etc ( ) Other (please specify):: Endovascular Aortic Repair *4) Does your centre offer vascular services?* ( ) Yes ( ) No *5) Who is performing aortic repair at your centre? [question only displayed if answer to question 4 is “yes”]* ( ) IRs alone ( ) Vascular and/or cardiovascular surgeons alone ( ) Multidisciplinary team of vascular and/or cardiovascular surgeons and IRs ( ) Angiologists/cardiologists alone ( ) Surgeons and angiologists/cardiologists *6) Which access for endovascular aortic repair is usually performed at your centre? (percutaneous vs surgical cut down) [question only displayed if answer to question 4 is “yes”]* ( ) Surgical cut down by vascular and/or cardiovascular surgeons ( ) Surgical cut down by multidisciplinary teams ( ) Percutaneously using closure devices by vascular and/or cardiovascular surgeons ( ) Total percutaneous aortic repair by multidisciplinary teams *7) Please indicate below the involvement of IRs in endovascular aortic repair at your centre by selecting all levels that apply* [ ] Level 0: Imaging and image interpretation – IR only or MDT [ ] Level 1: Measurement and prosthesis sizing – IR only or MDT [ ] Level 2: attending every procedure as equal operator in an MDT, like switching first and second operator – done in the IR unit or vascular surgeons’ operating room [ ] Level 3: procedures are done exclusively by IRs *8) Are you involved in endovascular aortic repair?* ( ) Yes ( ) No *9) Do you perform the following procedures? Please select all that apply: [question only displayed if answer to question 8 is “yes”]* [ ] EVAR [ ] TEVAR [ ] fEVAR/BEVAR [ ] Arch endovascular repair [ ] Embolization (either pre-emptive and/or endoleak) *10) What are the reasons you are not involved in aortic repair? Please select all that apply: [question only displayed if answer to question 8 is “no”]* [ ] Performed by other specialities [ ] Financial reasons [ ] Lack of time [ ] No interest [ ] Lack of workforce [ ] Lack of training [ ] Lack of industry support [ ] Hospital administration decision	*11) Do you offer training to junior IRs and/or trainees in endovascular aortic repair? [question only displayed if answer to question 8 is “yes”]* ( ) Yes ( ) No *12) Why do you not offer training to junior IRs and/or trainees in this field? [question only displayed if answer to question 11 is “no”]* ( ) No interest ( ) No training material ( ) Other—please specify: *13) Do you expect growth in your endovascular aortic practice in the next 5–10 years? [question only displayed if answer to question 8 is “yes”]* ( ) Yes ( ) No ( ) Undecided *14) Why do you not expect growth in your endovascular aortic repair practice in the next 5–10 years? [question only displayed if answer to question 13 is “no”]* [ ] Lack of workforce [ ] Lack of interest [ ] Lack of training [ ] Turf competition *15) Have you been trained in endovascular aortic repair? [question only displayed if answer to question 8 is “no”]* ( ) Yes ( ) No *16) What could support the growth of IR practice in the field of endovascular aortic repair in the next 5–10 years?* *Please select all answers you consider relevant:* [ ] Dedicated CIRSE fellowship [ ] Training [ ] Online courses / webinars [ ] Qualification to have clinical practice [ ] Increase the number of IR practitioners [ ] Further technology refinement including lower profile devices EBIR-ES and Endovascular Subcommittee *17) Are you an EBIR holder?* ( ) Yes ( ) No *18) Are you a CIRSE-certified Endovascular Specialist (EBIR-ES)? [question only displayed if answer to question 17 is “yes”]* ( ) Yes ( ) No *19) Would you be interested for being considered as faculty for the CIRSE IDEAS programme?* ( ) Yes ( ) No *20) If you are interested to be considered as future faculty for the CIRSE IDEAS, please indicate your e-mail address below. [question only displayed if answer to question 19 is “yes”]* 21) Generally speaking, which tools / documents / guidelines that are not yet available would help you in your daily endovascular work? [open text]

## Results

**Fig. 1:**
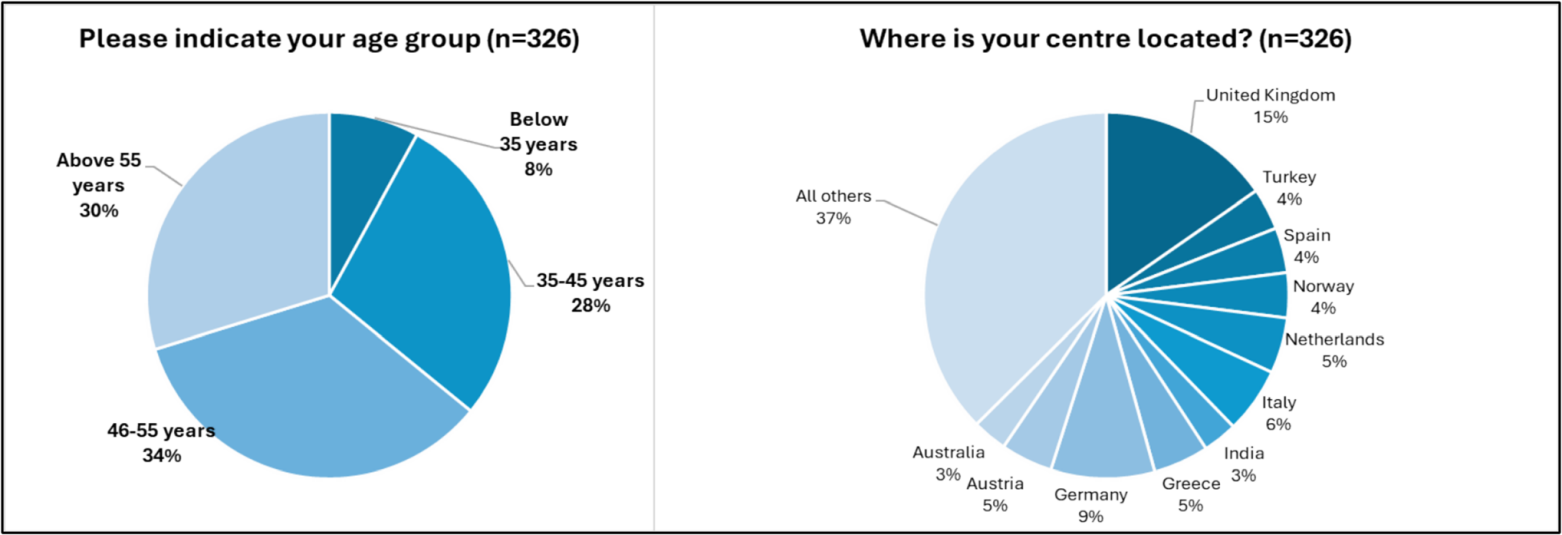
Responses to main demographic questions

The total number of respondents was 326, corresponding to a response rate of 4.5%. Most participants were from European countries. The distribution across age groups was approximately even (see Fig. 1). Almost 60% of participants were EBIR holders, but only a third of them were EBIR-ES qualified. More than 60% of respondents work in teaching and/or academic centres, and almost all participants have vascular services in their base hospital.

**Fig. 2:**
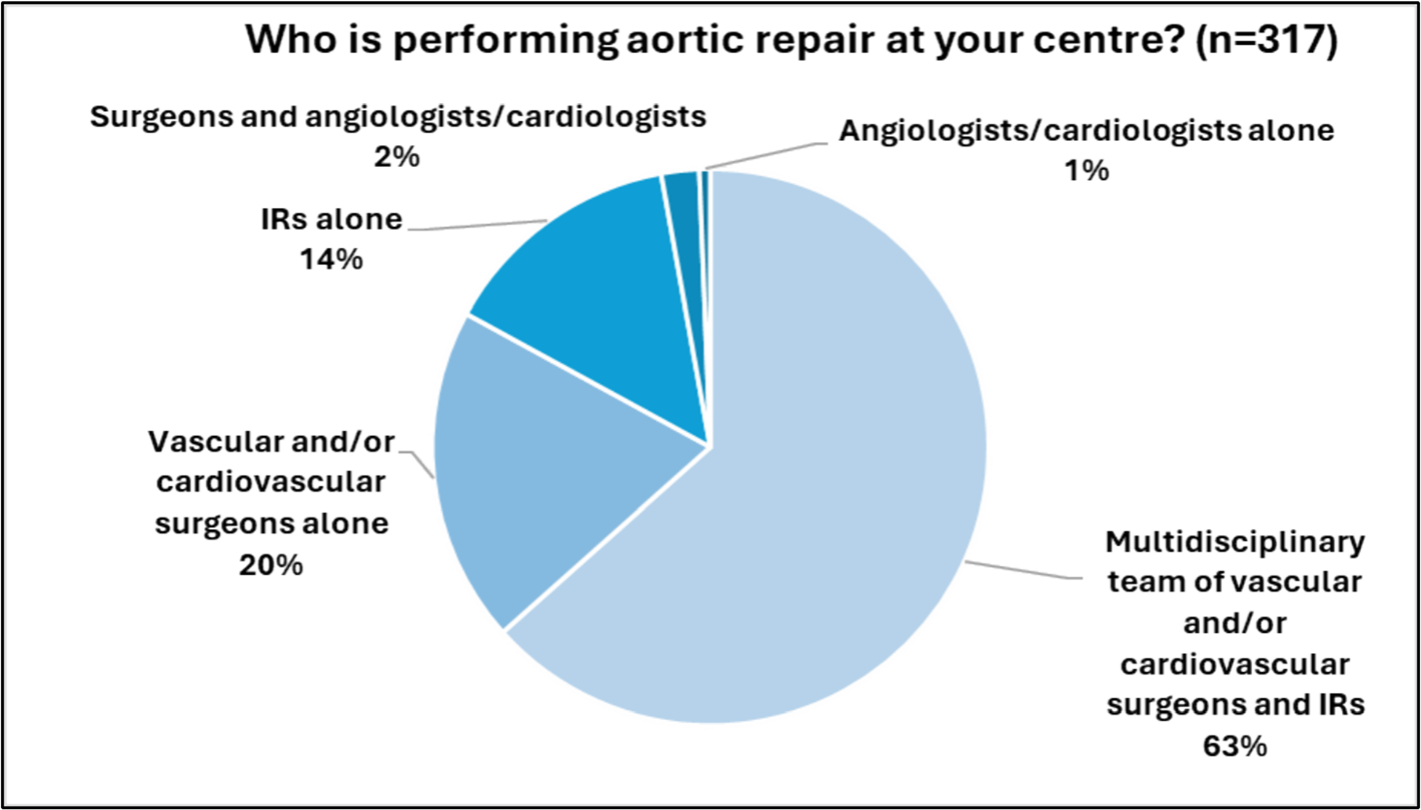
Disciplines involved in aortic repair

Two-thirds of respondents reported their involvement in endovascular aortic services within multidisciplinary teams, while 20% indicated that vascular or cardiovascular surgeons deliver aortic services. More than half of the survey participants stated that they are equal partners in performing the procedures (see Fig. 2), whereas 27% indicated that they mainly act as the primary operators.

**Fig. 3:**
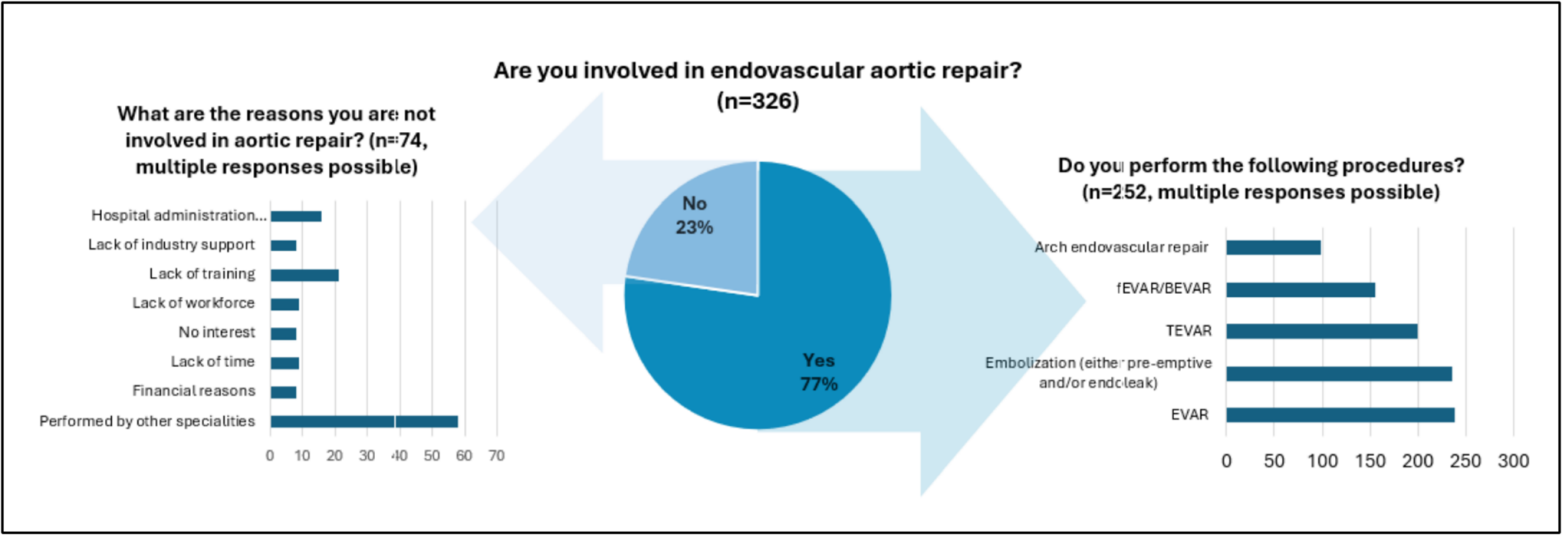
Involvement in endovascular aortic repair

**Fig. 4:**
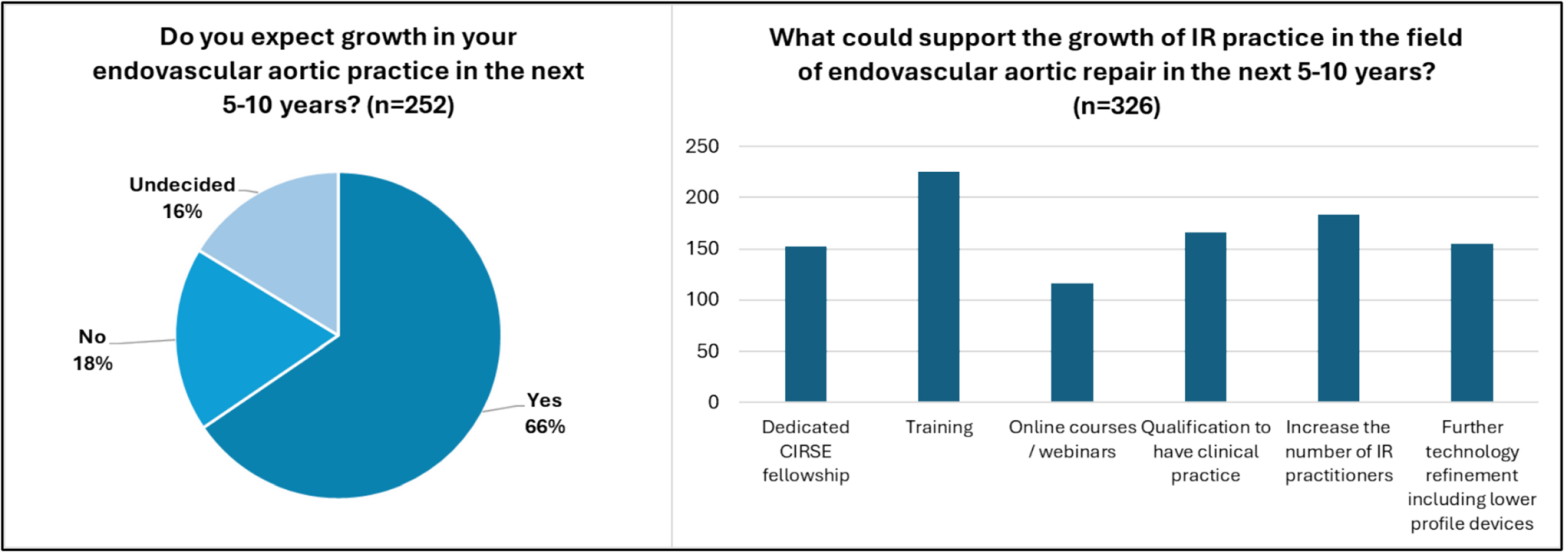
Expected growth and required support for endovascular aortic repair in the coming years

Concerning the types of endovascular aortic procedures, EVAR and embolisation of endoleaks were the most frequently performed, followed by FEVAR and TEVAR (see Fig. 3). Most endovascular aortic procedures were performed using percutaneous access by combined IR–vascular surgery teams or vascular surgeons alone. For those respondents who reported no involvement, the reasons were attributed to the procedure being carried out by a different speciality, followed by a lack of training or decisions made by hospital management (see Fig. 4).

Most survey participants involved in endovascular aortic repair expect a growth in endovascular work, with 64% predicting an increase. Regarding training, interestingly, most respondents involved in endovascular aortic repair also reported that training is offered. Only 13% believed that the material is inadequate, and there is a deficiency of proper fellowship training or other learning opportunities. In the section on additional comments, several participants mentioned that guidelines by CIRSE could help improve both the quality of care and the profile of interventional radiology. Additionally, improved equipment, more training resources, establishing clinical practice, and a more defined IR speciality have been identified as further tools or needs that could enhance the IR role in the aortic endovascular field.

## Discussion

This survey has shown that many CIRSE members are currently involved in the management pathway of aortic patients. This is contrary to a growing perception among several specialities and entities, including industry, that the aortic field is outside the IR arena.

Although it is probably true that EVAR procedures are increasingly no longer performed solely by interventional radiologists, the survey has shown that about 63% of respondents still carry out these procedures within a multidisciplinary team, and collaborative models appear to work well with excellent outcomes. The respondents also demonstrate a significant interest in either continuing or expanding their role in endovascular aortic repair.

Interestingly, 64% of respondents who are involved in endovascular aortic repair opine that growth is expected in this area. The respondents believe that supporting IR to expand their involvement in aortic practice can be achieved through training, a dedicated CIRSE fellowship, increasing the workforce, CIRSE guidelines, and further device refinements. These suggested steps or measures can be realised by collaborative actions between CIRSE and national IR societies as well as individual initiatives.

Regarding those respondents who do not anticipate growth in practice (around 35%), turf competition is the most common cause of limited expansion, followed by hospital management decisions. The latter point underscores the well-recognised lack of visibility of interventional radiology (IR) and the limited understanding among the public and fellow professionals concerning IR skills, as well as the role and potential benefits that the speciality could provide. Although local politics primarily influence the IR department’s relationship with hospital management, the authors recommend that members utilise CIRSE resources, certifications, and tools to advocate proactively with management and the public, thereby boosting IR’s profile and demonstrating its vital role. Workforce shortages, lack of interest, workload burden of diagnostic radiology, and inadequate training are cited as factors restricting IR involvement. These challenges are well-recognised in IR and are not limited to the aortic field but extend to other body systems as well. The recent global IR consensus statement clearly states that having speciality status and following clinical practice can help to address several adverse factors that hinder the growth of IR [10]. Endovascular aortic interventions extend far beyond the technical insertion of stents. Interventional radiologists have acknowledged expertise in pre-procedural planning; dealing with procedural complications, and reinterventions that require high technical skills. This is reflected in the survey results, which demonstrated IR involvement in a wide range of aortic-related procedures, especially embolization of endoleak.

Training is the cornerstone in maintaining and expanding future aortic practice. Although detailed steps and actions to support training were not surveyed, the authors consider that the CIRSE Academy, ESIR workshops, structured simulation, and further additions to IR curricula to include human factors training can be effective measures to equip future generations with the necessary skills and confidence.

Several freehand comments emphasized the importance of developing practice guidelines by CIRSE. This not only helps guide practitioners but also elevates the speciality’s profile.

Limitations of this survey are the relatively low number of responses and the potential for bias among the respondents. However, we believe that the responses by the participants are valid and are in keeping with current practice by IRs in aortic therapy.

## Conclusion

This survey confirms that interventional radiologists (IRs) continue to play a significant role in endovascular aortic procedures. There are challenges and opportunities to enhance and reinforce the role of IR in endovascular aortic aneurysm procedures to improve patient care. It is important that CIRSE, in collaboration with national societies, facilitates and encourages the clinical role of IR practice in standard and complex EVAR to best guide future developments for optimal patient management in aortic disease.
